# Retrospective evaluation of the use of small‐bore wire‐guided catheters for the management of peritoneal effusion in cats and dogs

**DOI:** 10.1111/vec.13265

**Published:** 2022-11-30

**Authors:** Jilli Crosby, Karen Humm, Simon D. Cook

**Affiliations:** ^1^ Department of Clinical Science and Services The Royal Veterinary College Hatfield UK; ^2^ Present address: Fitzpatrick Referrals, Halfway Lane, Godalming, GU7 2QQ

**Keywords:** ascites, complications, drainage

## Abstract

**Objective:**

To describe the use of small‐bore wire‐guided catheters in the management of peritoneal effusion in cats and dogs and to detail any associated adverse events.

**Design:**

Retrospective study.

**Setting:**

University teaching hospital

**Animals:**

Forty‐five client‐owned animals that had peritoneal catheters placed for management of peritoneal effusion between July 2010 and June 2021.

**Interventions:**

None.

**Measurements and Main Results:**

Forty‐five cases were included (25 dogs and 20 cats). Twenty‐eight animals had the catheter placed to aid management of a uroabdomen, 8 of which recovered without surgical management, 11 had the catheter placed to allow autotransfusion of hemoabdomen, 3 had peritonitis, and 3 had ascites secondary to cardiac disease. Twenty‐seven cases (15 dogs and 12 cats) received sedation (*n* = 24) or local anesthesia alone (*n* = 3) to facilitate catheter placement, and 6 cases had the catheter placed while under general anesthesia. Median length of catheter persistence was 24 hours (range: 2–144 h). The most common adverse events reported were impaired drainage (*n* = 7) and leakage at the insertion site (*n* = 4).

**Conclusions:**

Peritoneal catheters can be inserted percutaneously for management of peritoneal effusion. Indications include stabilization and conservative management of uroabdomen, and autotransfusion. They can often be placed with minimal or no sedation and adverse events appear infrequent in occurrence.

**Abbreviation**
PRBC: packed red blood cells

## 1 INTRODUCTION

Peritoneal drainage catheters are frequently placed surgically in cats and dogs for postoperative management of septic peritonitis,^1^ uroabdomen,^2^ and other inflammatory or exudative states.^3^, ^4^, ^5^ In people, the use of percutaneously placed peritoneal drainage catheters has been described for management of urogenital tract rupture,^6^, ^7^ malignant ascites,^8^ postsurgical peritoneal fluid,^9^, ^10^, ^11^, ^12^ post‐laparoscopy peritoneal gas,^13^ and intraabdominal or pelvic abscesses.^14^

In veterinary medicine, percutaneously placed small‐bore guidewire‐inserted chest drains are predominantly used for the management of pleural space disease^15^ including pyothorax,^16^ but their use in the management of pericardial effusions has also been described.^17^ These drains are placed by a modified Seldinger technique and generally do not require general anesthesia to be placed; they can be placed in conscious animals under sedation, with local anesthesia.^15^ They can also be placed into the peritoneal cavity of dogs and cats, but this technique has not previously been described in the literature.

Various techniques for performing abdominocentesis have been described including with needles, intravenous catheters, and peritoneal dialysis catheters.^18^, ^19^ Abdominocentesis can be considered therapeutic when used to improve patient comfort or alleviate clinical signs associated with ascites such as tachypnea^20^ or inappetence,^21^ or where removal of the fluid carries other clinical benefits such as limiting peritonitis or preventing re‐absorption of nitrogenous waste products, as in the case of uroabdomen.^22^ If repeated or ongoing drainage is deemed clinically necessary, having a peritoneal catheter secured in place may be advantageous, allowing drainage to be performed by a single suitably trained person.

This retrospective study aimed to describe the use of percutaneously placed small‐bore wire‐guided catheters for the management of peritoneal effusion in cats and dogs, detailing indications, duration of use, and any adverse events seen.

## 2 MATERIALS AND METHODS

The electronic medical case record and diagnostic imaging databases of the Queen Mother Hospital for Animals, at the Royal Veterinary College, were searched for cases in which a percutaneously placed, wire‐guided catheter^a^ was inserted into the peritoneal cavity between July 2010 and June 2021. Search terms “MILA AND peritoneal,” “abdominocentesis AND MILA,” “peritoneal drain,” and “peritoneal catheter” were used, and cases were reviewed for inclusion by all authors. Exclusion criteria were incomplete medical records, the use of different styles of catheter, and use of the catheter for other purposes than peritoneal fluid management. Data collected for each case included signalment, weight, reason for placement of the peritoneal catheter, sedative or anesthetic drugs used to aid placement of the peritoneal catheter, amount of fluid drained via the catheter, any adverse events associated with placement or maintenance of the catheter, antimicrobial use, length of catheter persistence, and reason for removal of the catheter.

## 3 STATISTICAL METHODS

All continuous data were assessed for normality by histogram inspection and descriptive data calculated as appropriate using commercially available software.^b^ Mean (± standard deviation) is presented for normally distributed variables and median (range) for skewed data.

## 4 RESULTS

**FIGURE 1:**
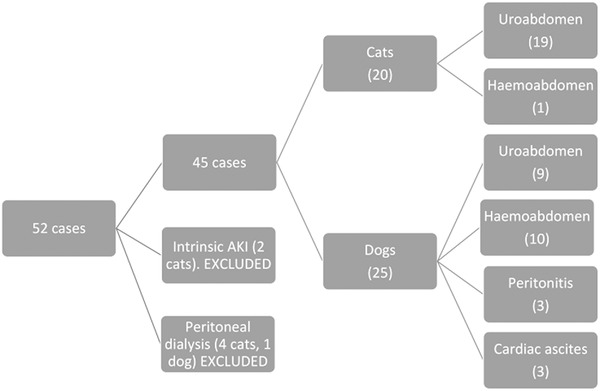
Flow chart detailing case recruitment and use of the peritoneal catheters

Fifty‐two cases where a percutaneously placed, wire‐guided peritoneal catheter was placed were identified. Five cases (4 cats and 1 dog) were excluded as the catheter was placed in order to facilitate peritoneal dialysis, not to manage peritoneal effusion. Two cases were excluded as they had peritoneal catheters placed to manage suspected uroabdomen; however, they were subsequently diagnosed with intrinsic acute kidney injury. Forty‐five cases remained of which 25 were dogs and 20 were cats. (Figure 1)

Dog breeds represented were crossbreeds (8), Labrador Retrievers (4), Bulldogs (2), and 1 each of the following: Beagle, Bernese Mountain Dog, Bichon Frise, Doberman Pinscher, English Setter, German Shepherd Dog, Golden Retriever, Irish Terrier, Manchester Terrier, Springer Spaniel, and Staffordshire Bull Terrier. Ten were female (7 of which were neutered [70%]) and 15 were male (9 of which were neutered [60%]). The median age was 98 months (range: 6–165 months).

Cat breeds represented were domestic short hair (12), British short hair (4), Bengal (2), and 1 each of domestic long hair and Siamese. Eight were female (6 of which were neutered [75%]) and 12 were male (10 of which were neutered [83%]). The median age was 48 months (range: 6–146 months).

Twenty‐eight of the 45 cases were diagnosed with uroabdomen, 11 cases with hemoabdomen, 3 cases with peritonitis, and 3 cases had ascites secondary to congestive heart failure (Figure 1).

Eighteen (8 dogs and 10 cats) of the 45 cases received sedation to facilitate drain placement, 6 cases (all dogs) received just analgesia, 3 cases (all dogs) had the catheter placed under local anesthesia (lidocaine injected into the skin and muscle of the area of drain insertion), and 4 cases (2 cats and 2 dogs) were specifically noted to have the catheter placed consciously. Six cases (4 cats and 2 dogs) were fully anesthetized for other purposes when the peritoneal catheter was placed, and details were unavailable for 8 cases.

The sedatives used most frequently to facilitate catheter placement were midazolam and an opioid. Eleven of 24 cases were administered midazolam, which was combined with an opioid (methadone or fentanyl) in 4 cases, ketamine in 4 cases, ketamine and methadone in 1 case, or used alone in 2 cases. An opioid (fentanyl, methadone, or butorphanol) was used alone in 7 cases and was combined with local anesthesia in 1 case. Propofol or alfaxalone were added into sedation protocols in 4 cases and medetomidine was used adjunctively in 3 cases. No notes were available on the sedation protocol in 3 cases.

The peritoneal catheters were all placed percutaneously using a modified Seldinger technique as previously described—through the ventral abdominal wall with the patient in lateral recumbency.^17^ The insertion site was not fixed and was guided by the ultrasonographic identification of a volume of peritoneal fluid. All catheters were 14 gauge, 20 cm chest drains with fenestrations either to 4 cm or to 8 cm. Where the length of fenestration was detailed, those fenestrated to 4 cm were placed in animals with a median weight of 4.5 kg (range: 2.7–50 [*n* = 9]) and those fenestrated to 8 cm were placed in animals with a median weight of 15.9 kg (range: 3.5–35.6 [*n* = 10]).

### 4.1 Uroabdomen

Twenty‐eight cases had a peritoneal catheter placed to aid the management of uroabdomen. Of these, 19 were cats and 9 were dogs. The etiology of uroabdomen was iatrogenic in 19 cases: after urinary tract surgery (*n* = 6), cystocentesis (*n* = 3), manual bladder expression (*n* = 2), and in association with management of urethral obstruction in 8 cases (after attempted urethral catheterization [*n* = 4] or cystocentesis [*n* = 4]). The etiology was blunt trauma in 5 cases, neoplastic in 1 case, and unknown in 3 cases. The purpose of the peritoneal catheters was to provide urinary diversion and to manage hyperkalemia that was present in 20 out of the 28 cases.

Seventeen of the 28 cases had peritoneal catheters placed prior to definitive surgical management of the uroabdomen—performed on the same day (*n* = 7), the following day (*n* = 8), or 2 days after catheter placement (*n* = 2). Details of fluid retrieval were available for review in 11 of the 17 cases. A median total volume of 21.0 ml/kg (range: 0–74.6 ml/kg) was yielded on initial drainage. The peritoneal catheter was drained once in 4 cases, and every 2–8 hours until catheter removal in 7 cases yielding a median fluid production rate of 3.43 ml/kg/h (range: 0–25.6 ml/kg/h). Two cases had a urine closed collection system attached aseptically to the peritoneal catheter but specific details were unavailable for the remaining cases. The peritoneal catheters were in situ for a median of 12 hours (range: 2–48 h) and were all removed at the time of surgery.

Eleven of the 28 uroabdomen cases did not undergo surgical management and the catheters were maintained in place for a median of 34 hours (range: 6.5–144 h). In 7 of these 11 cases, catheter production details were available for review and a median total volume of 32.6 ml/kg (range: 1.08–89.9 ml/kg) was removed on initial drainage. Subsequent drainage revealed a mean rate of fluid retrieval of 1.57 ml/kg/h (±3.38) and drainage took place every 2–8 hours. Two cases had a closed collection system attached to the peritoneal catheter but specific details were unavailable for the remaining cases. Catheters were removed due to cessation of urinary tract leakage (*n* = 8) (confirmed by positive contrast radiography study in 3 cases), cardiopulmonary arrest (*n* = 2), and euthanasia (*n* = 1). In the 8 cases that were successfully managed without surgery, the site of urine leakage was found to be the bladder (*n* = 4), the ureter (*n* = 1), or the urethra (*n* = 1). Leakage sites were not documented in 2 cases.

### 4.2 Hemoabdomen

Eleven peritoneal catheters were placed in cases with hemoabdomen. Of these, 10 were dogs and 1 was a cat. The cause of hemoabdomen was neoplasia in 8 cases (3 splenic masses, 1 adrenal mass, and 3 hepatic masses), blunt trauma in 1 case, uncharacterized coagulopathy in 1 case, and post‐ovariohysterectomy hemorrhage in 1 case.

All 11 cases had a peritoneal catheter placed for blood retrieval and autotransfusion purposes; autotransfusions were performed once in 8 cases, twice in 1 case, and 3 times in 1 case, and 1 case was euthanized before autotransfusion could be performed. The retrieved blood was processed with a cell salvage device^c^ prior to autotransfusion of packed red blood cells (PRBC) (*n* = 9), or was directly autotransfused as whole blood collected from the drain into syringes with anticoagulant citrate phosphate dextrose adenine solution (*n* = 2). In the 8 cases where it was documented, the median total volume of blood retrieved from the abdomen via the peritoneal catheter was 35.9 ml/kg (range: 17.0–71.3), and the median volume of PRBC or whole blood administered during autotransfusion was 16.5 ml/kg (range: 12.0–25.3).

Six cases underwent surgical correction of the hemorrhage, which took place on the same day as peritoneal catheter placement (*n* = 3), the following day (*n* = 2), or 3 days after catheter placement (*n* = 1). Of these cases, 4 had the peritoneal catheter removed during surgery, and 2 cases had the catheter removed within 24 hours of catheter placement, and after a single drainage. Four cases were euthanized with the peritoneal catheter still in place after evidence of neoplasia was identified on computed tomography. One case, with the uncharacterized coagulopathy, was successfully managed medically and had the peritoneal catheter removed within 24 hours and after a single drainage. The median length of catheter persistence was 24 hours (range: 5–48 h).

### 4.3 Peritonitis

One dog had a peritoneal catheter placed to aid the medical management of spontaneous bacterial peritonitis with small volume effusion. The catheter was used for drainage and then lavage on 2 occasions, being connected to a negative‐pressure closed collection system in between saline instillations. On initial catheter placement, 0.1 mg/kg fluid was removed but drainage was positional and the catheter was removed after 24 hours. The dog recovered and was discharged successfully.

Two dogs had peritoneal catheters placed to manage postoperative effusions. One had the catheter placed as an intraoperatively placed, Jackson–Pratt drain was nonfunctional (after surgery to correct gastric dilation and volvulus). The peritoneal catheter was also nonfunctional and fluid was noted to continuously leak from the insertion sites of both the Jackson–Pratt drain and the peritoneal catheter. The peritoneal catheter was in place for less than 24 hours before the dog was euthanized. The other case had a peritoneal catheter placed to manage a large volume, nonseptic effusion and improve patient comfort after surgical repair of a perforated duodenal ulcer. This catheter was in place for 120 hours before the patient was euthanized due to a suspected pulmonary thromboembolism.

### 4.4 Cardiac ascites

Three dogs had a peritoneal catheter placed for drainage of ascites associated with right‐sided congestive heart failure. On placement, 43.2, 206.0, and 327.3 ml/kg of fluids were respectively drained and the catheters were then removed immediately after drainage.

### 4.5 Antimicrobials

Ten out of 45 cases had complete records detailing antimicrobial prescription during hospitalization. Eight had uroabdomen and 2 had septic peritonitis. In 4 of these cases, the antimicrobials were prescribed to treat a septic abdominal effusion and none were suspected to have developed the infection due to abdominal catheter placement; the abdominal catheters were placed as part of the management protocol. In the other 5 cases, antimicrobials were prescribed for the treatment of urinary tract infection (*n* = 2), aspiration pneumonia (*n* = 1), cellulitis (*n* = 1), and an unclear reason (*n* = 1). Of 12 peritoneal fluid samples cultured, 8 were negative and none of the 4 positive cultures were repeated at a later date.

### 4.6 Adverse events

The most frequently reported adverse events were difficulties draining, or failure to retrieve fluid from the catheter (*n* = 6) (Table 1). This was successfully managed in 3 cases (2 uroabdomen cases and 1 congestive heart failure case) where the catheter was either flushed with sterile saline (*n* = 1), repositioned within the abdomen (*n* = 1), or had a closed collection system attached (*n* = 1). In 2 cases (1 uroabdomen and 1 septic peritonitis case), the peritoneal catheter was unproductive despite the continued presence of peritoneal effusion, and the catheter was removed. In 1 uroabdomen case, a closed collection system was initially attached to the catheter at the time of placement; however, drainage was reported to be poor and manual drainage was performed successfully.

**TABLE 1:** Adverse event frequencies reported per indication in a group of dogs and cats that had a small‐bore wire‐guided catheter placed for management of peritoneal effusion

	Adverse events			
	Draining difficulty	Leakage	Local discomfort	Difficulty placing
Uroabdomen	4	1	2	1
Hemoabdomen	0	1	0	0
Peritonitis	1	0	0	0
Cardiac ascites	1	1	0	0

The second most frequent adverse event was leakage around the catheter insertion site in 3 cases (1 case each of uroabdomen, hemoabdomen, and ascites due to cardiac disease). The catheter was patent and functional in 3 of these cases and the leakage was managed as part of the patients’ nursing care.

In 2 cases of uroabdomen, it was suspected that the presence of the peritoneal catheter was associated with local discomfort due to pain on abdominal palpation in 1 case, and aggression on abdominal palpation in the other case. However, this could not be definitively attributed to the catheter due to the underlying disease, patient temperament, and the presence of a local surgical site.

Difficulty placing the peritoneal catheter was noted in 1 case where the catheter was only successfully placed on the fourth attempt. It is not clear in the clinical notes why the first 3 attempts were unsuccessful.

## 5 DISCUSSION

This study describes the most frequent indications for percutaneous placement of a peritoneal catheter at this referral hospital: management of animals with uroabdomen and hemoabdomen. Patients presenting with uroabdomen often benefit from medical stabilization prior to definitive treatment.^23^ The treatment options for these patients depend on the site of urogenital tract rupture and the presence or absence of ongoing leakage.^4^, ^24^, ^25^, ^26^ The most frequent indication for placement of a peritoneal catheter in this study was stabilization of patients with uroabdomen (*n* = 28), but in 8 of these, it provided successful urinary diversion, along with urinary catheterization, and served as definitive treatment.

The use of peritoneal catheters to facilitate autotransfusion has not been previously described. Typically, collection of blood for autotransfusion would be performed by needle paracentesis using a needle or butterfly catheter,^27^ or intraoperatively using a suction device^28^ or syringe.^29^ Preoperative collection of peritoneal blood can allow patient stabilization without reliance on autologous blood products (which may be expensive or difficult to source). The use of a peritoneal catheter may enable faster drainage compared to needle paracentesis and it could be hypothesized that hemolysis due to shear injury would be decreased. It may also aid the maintenance of sterility as the need for repeated percutaneous puncture would be removed.

In this study, 3 catheters were placed to manage fluid accumulation in dogs with peritonitis. This practice is described in people^10^, ^11^, ^12^ and in veterinary medicine for postoperative care in animals,^1^, ^30^, ^31^ but a benefit to their use cannot be inferred from this study. The peritoneal catheters in these cases seemed to be used to retrieve existing inflammatory or infective fluids where that was deemed beneficial by the attending clinicians.

Another indication for the placement of a peritoneal catheter is to manage large‐volume ascites and the associated increase in intraabdominal pressure and discomfort.^32^ Symptoms such as respiratory difficulty, abdominal pain, and restricted mobility have been reported in people due to increased abdominal pressure.^10^ Therapeutic abdominocentesis has been reported and advocated to manage ascites caused by neoplasia, hepatic disease, and cardiac disease in dogs.^3^, ^4^, ^5^, ^20^, ^21^ Once a peritoneal catheter is placed, ascites can be drained by 1 person, decreasing personnel requirements when a large volume of fluid is present.

Of the patients that received analgesia, sedation, or anesthesia to aid drain placement (33/45 [73.3%]), many required only mild sedation or analgesia. This suggests that in an emergently or critically unwell patient, chemical restraint is not always required for this procedure, meaning that these catheters may be rapidly placed with minimal cardiovascular or respiratory compromise. These findings are similar to studies evaluating the use of small‐bore wire‐guided percutaneous catheters in the management of pleural space disease^17^ and of pericardial effusion.^19^

The most frequent adverse event was impaired drainage from the peritoneal catheter (*n* = 6 [13.3%]), which was resolved by routine troubleshooting in most cases. A similarly low rate (13.8%) is reported in percutaneously placed drains in people, with reported adverse events including several not documented in this study (drain infection, organ puncture, bleeding) and some that were (fluid leakage at the catheter insertion site and catheter occlusion).^34^, ^35^ Infection of catheter insertion sites was not appreciated in this study, likely due to the relatively short duration of catheter persistence. In people, subcutaneous tunnel infections and bacterial peritonitis in association with the presence of a peritoneal catheter have been reported to occur after approximately 3–5 weeks.^35^, ^36^, ^37^ A veterinary study investigated the use of surgically placed thoracostomy tubes in 8 healthy dogs and noted that 6 developed pyothorax within 7 days.^38^ These thoracostomy tubes had a diameter of 20 Fr and were therefore significantly wider than the 14‐g catheters used in this study. The use of small‐bore catheters is recommended in people due to lower rates of infection, as well as fewer insertional complications, when compared to larger bore drains.^17^ Further research into rates of insertion site infection and peritonitis associated with the use of small‐bore peritoneal catheters could be warranted if they are used longer term.

Leakage at the insertion site has been reported to occur more frequently in percutaneously placed catheters (20.5%) when compared with surgically placed catheters (6.8%) in people.^38^ This study has demonstrated that 6.7% of cases leaked, which can be considered a reasonably low frequency. Furthermore, a similar rate of failure of placement was observed in this study (2.2%) compared to that in people (2.6%).^38^

The retrospective nature of this study means that it is not possible to accurately capture and describe the clinical decision‐making process used in the management of cases described. The placement and removal of the peritoneal catheters were at the clinician's discretion, and it is also possible that there may have been unreported adverse events. It was often not possible to determine the technique used when draining the peritoneal catheters. Future questions to interrogate would include whether the peritoneal catheters expedite stabilization or confer a benefit in patients with uroabdomen if urinary diversion is achieved by other means, such as urethral catheterization. Infectious complication rates are perhaps best assessed by prospective evaluation of peritoneal catheters indwelling for longer periods than those described here. Direct comparison of hemoabdomen blood collection by peritoneal catheter with other techniques, including evaluation of sampling artefact, volume collected, and ease of collection, may also be considered.

In conclusion, the placement of peritoneal catheters may be considered for single timepoint or ongoing removal of peritoneal effusions. In cases where repeated abdominocentesis or continuous drainage may be required, or where patient temperament is challenging, the placement of an indwelling peritoneal catheter may improve patient comfort as well as allow more frequent and complete drainage of the peritoneal cavity without chemical restraint. Although adverse events were noted associated with percutaneous peritoneal catheter placement and use, these were easily managed. These catheters offer a minimally invasive therapeutic option in cases of uroabdomen that are amenable to conservative treatment, or for autotransfusion purposes.

## CONFLICT OF INTEREST

The authors declare no conflict of interest.

## ENDNOTES

a Guidewire inserted chest tube; MILA International Inc., Florence, KY.

b Microsoft Excel; Microsoft, Washington, DC.

c C.A.T.S.; Fresenius Kabi AG, Bad Homburg, Germany.
